# Our Experience With Intractable Epistaxis After COVID-19 Nasopharyngeal Swab

**DOI:** 10.7759/cureus.65014

**Published:** 2024-07-20

**Authors:** Ahmed Shaikh, Rani Hammoud, Emad Al Duhirat, Adham Aljariri, Fatima Emam, Hamad Al Saey, Mansour Al Sulaiti, Shanmugam Ganesan

**Affiliations:** 1 Otolaryngology-Head and Neck Surgery, Hamad Medical Corporation, Doha, QAT; 2 Radiology, Hamad Medical Corporation, Doha, QAT

**Keywords:** nasopharyngeal, intractable epistaxis, sphenopalatine artery ligation, covid-19 swab, epistaxis

## Abstract

Introduction: Although a COVID-19 nasopharyngeal swab is a safe procedure routinely performed by healthcare providers, it can lead to complications that can be life-threatening. We present seven cases of intractable epistaxis following a nasopharyngeal swab that required sphenopalatine artery ligation. We aim to shed light on this life-threatening condition, emphasizing the importance of recognizing and mitigating such complications.

Materials and methods: This retrospective chart review involved cases of intractable epistaxis following a COVID-19 swab from January 2020 to June 2022. The patient's charts were reviewed for the location of the epistaxis and different intranasal and extranasal factors that could have led to it.

Results: Seven cases had intractable epistaxis following a nasopharyngeal COVID-19 swab. Six of the seven cases had a deviated nasal septum, and one case had an enlarged inferior turbinate. All patients had bleeding from the ipsilateral nasal structural abnormality. All patients underwent successful sphenopalatine artery ligation.

Conclusion: Our study highlights the significance of recognizing the potential risk of intractable epistaxis post-COVID-19 swabs and emphasizes the importance of comprehensive training programs to ensure the safe and effective execution of nasopharyngeal swab procedures.

## Introduction

Nasopharyngeal swabs using Q-tips have been the gold standard for the collection of specimens in the diagnosis of COVID-19 disease [1]. CDC guidelines for the diagnosis of SARS-CoV-2 recommend that diagnostic swabs be performed from the nasopharynx, oropharynx, mid-inferior turbinate, or anterior nares. However, the best diagnostic sensitivity was reported from nasopharyngeal swabs [1-3]. Many nasopharyngeal swabs are performed each day worldwide; the complications are rare, and the rate of complications reported in the literature ranges from 0.0012% to 0.026% [4,5]. The most commonly documented complications reported in the literature are retained swab sticks as a foreign body, epistaxis, and cerebrospinal fluid rhinorrhea [4-6]. Intractable epistaxis after COVID-19 swab is a rare complication, with very few documented cases in the form of case reports in the literature [1,7-9].

Intractable epistaxis, defined as persistent nosebleeds that are difficult to control despite initial medical management, presents a significant challenge in otolaryngology. It often necessitates advanced interventions beyond standard treatments, such as nasal packing or simple cauterization, and requires surgical intervention in most instances [10]. In this paper, we describe our experience and the clinical characteristics of seven cases of intractable epistaxis secondary to COVID-19 swab tests that underwent sphenopalatine artery ligation. Our goal is to shed light on this life-threatening condition, highlighting the crucial need for recognition and prevention of such complications.

## Materials and methods

Area of study

After receiving approval from the ethical committee of Hamad Medical Corporation, we conducted a retrospective descriptive study from January 2020 to June 2022. The aim was to investigate the clinical characteristics of patients who experienced intractable epistaxis following COVID-19 swabs.

Inclusion and exclusion criteria

Inclusion criteria included patients with intractable epistaxis following COVID-19 swabs. Intractable epistaxis is defined as bleeding not controlled with nasal packing and requiring control under general anesthesia with ipsilateral sphenopalatine artery ligation/cauterization. Exclusion criteria include patients with intractable epistaxis secondary to other causes and patients with incomplete medical charts. 

Data collection

We extracted clinical and demographic data from electronic medical records, such as age, gender, use of anticoagulants and antiplatelet (APT) agents, history of hypertension, smoking status, diabetes mellitus, location and laterality of bleeding, drop in hemoglobin level, requirement for blood transfusion, presence of septal deviation, types (according to Mladina's Classification*) and site of septal deviation, presence of ipsilateral inferior turbinate hypertrophy, and any concomitant procedures during the control of bleeding. 

Data analysis

Data analysis for the retrospective descriptive study was conducted using Microsoft Excel 2020. Descriptive metrics, including mean, standard deviation, and percentage, were calculated to summarize the clinical and demographics of the study.

## Results

We present seven patients who presented to the emergency department with severe nasal bleeding following nasopharyngeal swab testing for COVID-19. In all cases, anterior and posterior nasal packing failed to control the nasal bleeding, and eventually, they required control under general anesthesia with an ipsilateral sphenopalatine artery ligation. Table 1 summarizes the key clinical details of each case.

**Table 1:** The clinical characteristics of patients. AC: anticoagulants; APT: antiplatelet; HTN: hypertension; DM: diabetes mellitus; SD: septal deviation; ITH: inferior turbinate hypertrophy; SMD: submucosal diathermy of inferior turbinate; Hb: hemoglobin; post-op: postoperative. *Classified according to Mladina’s Classification. **Additional surgery to sphenopalatine artery ligation/cauterization.

Case	Age	Gender	Use of AC	Use of APT	HTN	Smoking	DM	Location of bleeding	Type of SD*	Site of bleeding	Bleeding same side as SD	ITH	Add-on surgery**	COVID status	Baseline Hb	Post-Op Hb	Drop in Hb	Required blood transfusion
1	38	Male	No	No	No	Yes	No	Sphenopalatine area just at the attachment of middle turbinate	Type V to the right	Right	Yes	No	Septoplasty	+ve	15.3	13.3	2	No
2	55	Female	No	No	No	No	No	Sphenopalatine area just at the attachment of middle turbinate	Type V to the right	Right	Yes	No	Septoplasty	-ev	13.2	8.7	4.5	Yes
3	47	Male	No	No	No	No	No	Sphenopalatine area just at the attachment of middle turbinate	Type III to the left	Left	Yes	Yes	Septoplasty + SMD	-ve	14.5	13	1.5	No
4	34	Male	No	No	No	Yes	No	Sphenopalatine area just at the attachment of middle turbinate	Type V to the right	Right	Yes	No	Septoplasty	-ve	15	13	2	No
5	62	Male	No	Yes	Yes	No	Yes	Sphenopalatine area just at the attachment of middle turbinate	Type V to the left	Left	Yes	No	Septoplasty	-ve	14.7	9.7	5	Yes
6	42	Male	No	No	No	Yes	No	Sphenopalatine area just at the attachment of middle turbinate	Type VI to the left	Left	Yes	No	Septoplasty	-ve	14.2	11.9	2.3	No
7	39	Female	No	No	No	No	No	Inferior turbinate at the posterior aspect	No deviation	Left	-	Yes	SMD	-ve	16	14.4	1.6	No

The mean age is 45.29 ± 9.32 (SD) years. Most patients were male, five (71.4%), and females accounted for two (28.6%). None of the patients were on anticoagulants, and one was on APT therapy. Hypertension (HTN) was present in one (14.3%) patient, two (28.6%) were smokers, and one had diabetes mellitus. Most bleeding was located in the sphenopalatine area at the middle turbinate attachment (85.7%), with 14.3% in the inferior turbinate at the posterior aspect. Septal deviation types included Type V in four patients (57.1%), Type III in one patient (14.3%), Type VI in one patient (14.3%), and no deviation in one patient (14.3%). Bleeding occurred on the same side as the septal deviation in 85.7% of cases. Inferior turbinate hypertrophy (ITH) was noted in 28.6% of patients. Additional surgeries to the sphenopalatine artery ligation/cauterization included septoplasty in five patients (71.4%), septoplasty with submucosal diathermy of inferior turbinate (SMD) in one patient (14.3%), and SMD alone in one patient (14.3%). One patient tested positive for COVID-19. Blood transfusions were required in two cases. The average baseline hemoglobin (Hb) was 14.27 g/dL (±SD: 0.82), postoperative Hb was 12.86 g/dL (±SD: 1.91), and the average drop in Hb was 2.23 g/dL (Table 2).

**Table 2:** Summary of the clinical characteristics. SD: standard deviation; AC: anticoagulants; APT: antiplatelet; SMD: submucosal diathermy of the inferior turbinate; ITH: inferior turbinate hypertrophy; SD: septal deviation; HTN: hypertension; Hb: hemoglobin; post-op: postoperative.

Characteristic	N
Mean age (years)	45.29 (±SD: 9.32)
Gender	
-Male	5 (71.4%)
-Female	2 (28.6%)
Use of (AC)	
-Yes	0 (0%)
-No	7 (100%)
Use of (APT)	
-Yes	1 (14.3%)
-No	6 (85.7%)
HTN	
-Yes	1 (14.3%)
-No	6 (85.7%)
Smoking	
-Yes	2 (28.6%)
-No	5 (71.4%)
Diabetes mellitus	
-Yes	1 (14.3%)
-No	6 (85.7%)
Location of bleeding	
-Sphenopalatine area at middle turbinate	6 (85.7%)
-Inferior turbinate at posterior aspect	1 (14.3%)
Type of SD	
-Type V	4 (57.1%)
-Type III	1 (14.3%)
-Type VI	1 (14.3%)
-No deviation	1 (14.3%)
Bleeding same side as SD	6 (85.7%)
ITH	2 (28.6%)
Add-on surgery	
-Septoplasty	5 (71.4%)
-Septoplasty+SMD	1 (14.3%)
-SMD	1 (14.3%)
Positive COVID status	1 (14.3%)
Required blood transfusion	2 (28.6%)
Mean baseline Hb	14.27 g/dL (±SD: 0.82)
Mean post-op Hb	12.86 g/dL (±SD: 1.91)

## Discussion

A nasopharyngeal PCR swab is a standard screening method for detecting the COVID-19 virus. It is generally safe and well tolerated and is usually performed by healthcare providers [1]. Although extremely rare, as in all procedures, complications have been reported in the literature [4,5,11]. These complications include retained swabs as a foreign body, epistaxis, and cerebrospinal fluid rhinorrhea [6,7].

Epistaxis post nasopharyngeal swab has been reported in approximately 16 cases in the literature, most of which were self-limiting or managed with nasal packing [1,6,7,9,11]. Nonetheless, there has been a report of potentially life-threatening epistaxis that required control with bipolar coagulation, artery ligation, and embolization [5,6,8]. In general, various factors can increase the risk of such complications, including ongoing nasal inflammation, anticoagulants, anatomical nasal variations, and improper nasal swab technique [6,7]. In our tertiary center, we have encountered seven cases of intractable epistaxis post-COVID-19 swab, all of which anterior and posterior nasal packs using either universal or Vaseline packs failed to control the bleeding. Eventually, the patients required control under general anesthesia with a unilateral sphenopalatine artery ligation where the maxillary sinus was identified, the flap was raised to identify the cristae ethmoidalis, and the sphenopalatine artery was cauterized and ligated.

**Figure 1:**
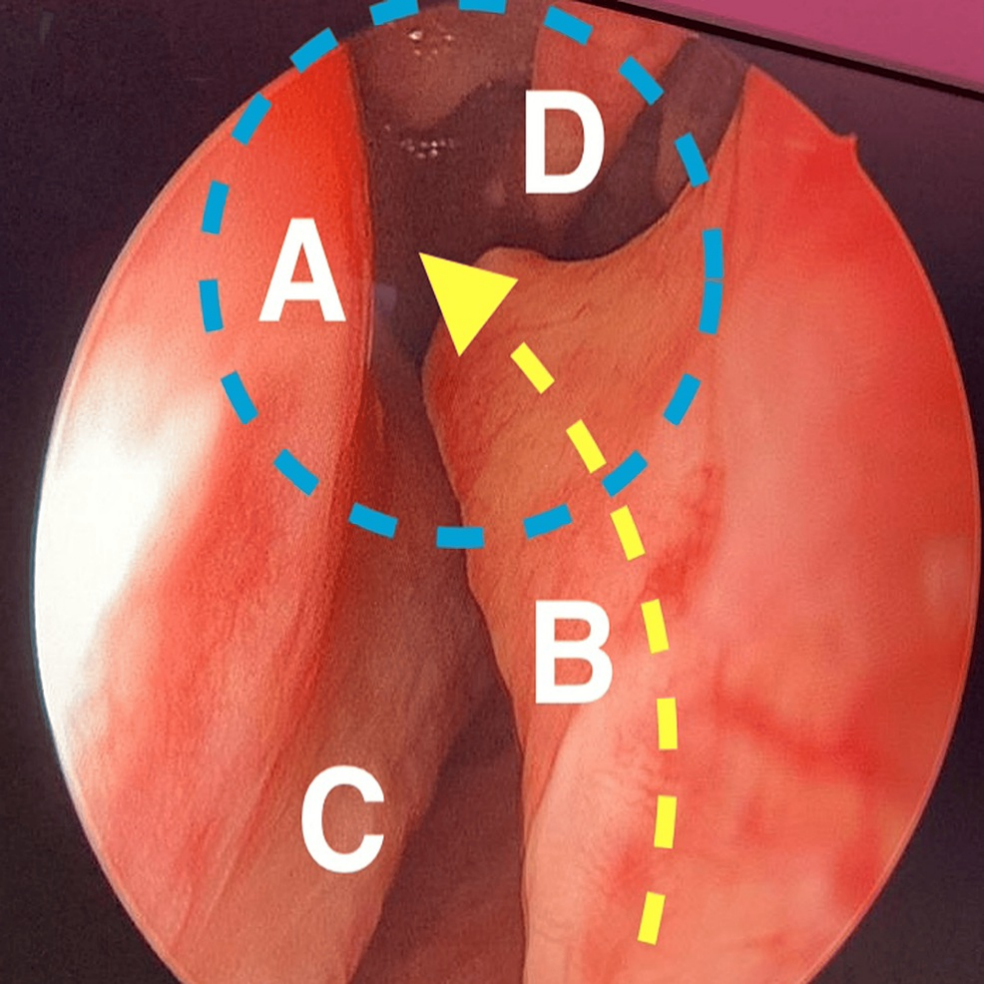
Endoscopic view of the right-side nasal cavity. A:  lateral nasal wall; B: nasal septal spur;  C: right inferior turbinate; D: right middle turbinate. Blue dotted circle: area of potential damage from the nasopharyngeal swab. Yellow dotted arrow: the improper trajectory of the nasopharyngeal swab.

**Figure 2:**
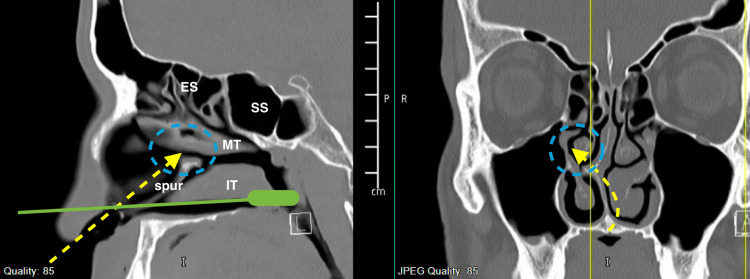
CT scan sinus. (Left) Sagittal cut. (Right) Coronal cut. CT: computed tomography; ES: ethmoid sinus; SS: sphenoid sinus; IT: inferior turbinate; MT: middle turbinate. Blue dotted circle: area of potential damage from the nasopharyngeal swab. Yellow dotted arrow: the improper trajectory of the nasopharyngeal swab. Green solid-line swab demonstrating the proper direction of nasopharyngeal swab.

Interestingly, all the patients had a nasal structural abnormality, with six having a septal deviation and one having an enlarged inferior turbinate. In addition, we observed that the site of the septal deviation was ipsilateral to the site of nasal bleeding. Intraoperative findings revealed nasal mucosal trauma just above and parallel to the septal spur at the lateral nasal wall corresponding to the sphenopalatine area just at the attachment of the ipsilateral middle turbinate, as seen in Figure 1. In addition to the sphenopalatine artery ligation, the nasal structural abnormality was also corrected intraoperatively in the same setting for access purposes and to improve patients' nasal breathing function; six patients had a septoplasty, and one patient had a submucosal diathermy of the inferior turbinate. Although computed tomography (CT) of the sinuses is not required in patients presenting with epistaxis, one patient had a previous CT in our system. Figure 2 shows the deviated nasal septum with the right-side septal spur along with the imagined improper trajectory of the nasopharyngeal swab. 

Regarding other factors that can potentially increase the risk of such complications, we had only one patient taking antiplatelet medications who also had a history of hypertension. Otherwise, the remaining patients had no other potential factors that could have increased the risk of bleeding following the nasopharyngeal swab. In all cases, bleeding was successfully controlled with ipsilateral sphenopalatine artery ligation, and patients were discharged after 24-48 hours of postoperative observation, and two patients required blood transfusion during their hospital stay. 

Although, in most cases, the nasopharyngeal swab is a safe and effective procedure to combat the pandemic, healthcare workers should be aware of the potential complications of the test. Poor familiarity with the structural nasal variations, such as deviated nasal septum, enlarged inferior turbinate, and nasal polyps, may lead to improper swab placement. Therefore, adequate training is crucial to mitigate such adverse effects. We encourage healthcare workers to perform the nasal swab on the ipsilateral side, which the patient reported as being most open during nasal breathing, and to abort it when faced with increased resistance or pain. 

This study has its limitations. The small sample size limits the generalizability of the findings. In addition, the absence of a control group makes it difficult to establish a causal relationship between the nasal factors and intractable epistaxis following nasopharyngeal swabs. 

## Conclusions

As the global healthcare community continues to combat the pandemic, the nasopharyngeal swab will remain a mainstay method for detecting COVID-19. Our experience underscores the importance of vigilance regarding potential complications, particularly in patients with nasal structural anomalies such as a deviated nasal septum.

This study highlights the significance of recognizing the potential risk of intractable epistaxis post-COVID-19 swabs and emphasizes the importance of comprehensive training programs to ensure the safe and effective execution of nasopharyngeal swab procedures. By enhancing familiarity with nasal anatomy and refining procedural techniques, we can further enhance the safety and efficacy of nasopharyngeal swabbing, ensuring its continued role as a cornerstone in pandemic control efforts while minimizing adverse patient outcomes.

## References

[1] Clark JH, Pang S, Naclerio RM, Kashima M (2021). Complications of nasal SARS-CoV-2 testing: a review. J Investig Med.

[2] Lee RA, Herigon JC, Benedetti A, Pollock NR, Denkinger CM (2021). Performance of saliva, oropharyngeal swabs, and nasal swabs for SARS-CoV-2 molecular detection: a systematic review and meta-analysis. J Clin Microbiol.

[3] Wölfl-Duchek M, Bergmann F, Jorda A (2022). Sensitivity and specificity of SARS-CoV-2 rapid antigen detection tests using oral, anterior nasal, and nasopharyngeal swabs: a diagnostic accuracy study. Microbiol Spectr.

[4] Föh B, Borsche M, Balck A, Taube S, Rupp J, Klein C, Katalinic A (2021). Complications of nasal and pharyngeal swabs: a relevant challenge of the COVID-19 pandemic?. Eur Respir J.

[5] Koskinen A, Tolvi M, Jauhiainen M, Kekäläinen E, Laulajainen-Hongisto A, Lamminmäki S (2021). Complications of COVID-19 nasopharyngeal swab test. JAMA Otolaryngol Head Neck Surg.

[6] Kim DH, Kim D, Moon JW, Chae SW, Rhyu IJ (2022). Complications of nasopharyngeal swabs and safe procedures for COVID-19 testing based on anatomical knowledge. J Korean Med Sci.

[7] Hakimi AA, Goshtasbi K, Kuan EC (2022). Complications associated with nasopharyngeal COVID-19 testing: an analysis of the MAUDE database and literature review. Am J Rhinol Allergy.

[8] Martin M, Greve J, Hoffmann TK, Hahn J (2022). Epistaxis requiring intervention after swab for SARS-CoV-2 (Article in German). Gesundheitswesen.

[9] Markussen DL, Hagen JE, Tvedt A, Steihaug OM (2021). Epistaxis after testing for COVID-19 (Article in Norwegian). Tidsskr Nor Laegeforen.

[10] Viehweg TL, Roberson JB, Hudson JW (2006). Epistaxis: diagnosis and treatment. J Oral Maxillofac Surg.

[11] Fabbris C, Cestaro W, Menegaldo A (2021). Is oro/nasopharyngeal swab for SARS-CoV-2 detection a safe procedure? Complications observed among a case series of 4876 consecutive swabs. Am J Otolaryngol.

